# Study of 320-Slice Dynamic Volume CT Perfusion in Different Pathologic Types of Kidney Tumor: Preliminary Results

**DOI:** 10.1371/journal.pone.0085522

**Published:** 2014-01-21

**Authors:** Chao Chen, Qi Liu, Qiang Hao, Bing Xu, Chao Ma, Huojun Zhang, Qianjin Shen, Jianping Lu

**Affiliations:** Department of Radiology, Changhai Hospital of Shang hai, The second Military Medical University, Shanghai, China; Institute of Automation, Chinese Academy of Sciences, China

## Abstract

**Objective:**

To investigate microcirculatory differences between pathologic types of kidney tumor using 320-slice dynamic volume CT perfusion.

**Methods:**

Perfusion imaging with 320-slice dynamic volume CT was prospectively performed in 85 patients with pathologically proven clear cell renal cell carcinoma (RCC) (*n* = 66), papillary RCC (*n* = 7), chromophobe RCC (*n* = 5), angiomyolipoma (AML) with minimal fat (*n* = 7), or RCC (*n* = 78). Equivalent blood volume (Equiv BV), permeability surface-area product (PS; clearance/unit volume = permeability), and blood flow (BF) of tumor and normal renal cortex were measured and analyzed. Effective radiation dose was calculated.

**Results:**

There was a significant difference in all three parameters between tumor and normal renal cortex (*P*<0.001). Equiv BV was significantly different between RCC and AML with minimal fat (*P* = 0.038) and between clear cell RCC and AML with minimal fat (*P*<0.001). Mean Equiv BV and BF were significantly higher in clear cell RCC than in papillary RCC (*P*<0.001 for both) and mean Equiv BV was higher in clear cell RCC than in chromophobe RCC (*P*<0.001). The effective radiation dose of the CT perfusion protocol was 18.5 mSv.

**Conclusion:**

Perfusion imaging using 320-slice dynamic volume CT can be used to evaluate hemodynamic features of the whole kidney and kidney tumors, which may be useful in the differential diagnosis of these four pathologic types of kidney tumor.

## Introduction

Ninety percent of all renal tumors come under five histologic types: clear cell carcinoma (or conventional carcinoma), which is the most common; chromophobe carcinoma; papillary carcinoma; and two common benign tumors, angiomyolipoma (AML) and oncocytoma [1]. AML is the most common benign tumor of the kidney; it comprises fat, smooth muscle, and abnormal blood vessels. The detection of intratumoral fat allows the radiologist to reliably and accurately identify AML [2], [3]. However, intratumoral fat cannot be visualized in an AML on cross-sectional imaging in cases of so-called AML with minimal fat [4]–[6]. These tumors can mimic renal cell carcinoma (RCC), leading to unnecessary surgery. In addition, RCCs account for 80–90% of all renal neoplasms [7]–[9], with multiple subtypes that differ in their histopathologic features, genetic expression pattern, and clinical behavior. Clear cell, papillary, and chromophobe are the most representative subtypes, accounting for 65–70%, 15–20%, and 6–11% of RCCs, respectively [10], [11]. These histopathologic entities differ in their prognosis and biologic behavior, as well as in their response to available therapies [12], [13]. Accurate subtyping of RCC by imaging is crucial for designing optimal treatment protocols and predicting prognosis [14].

Some investigators [6], [15]–[17] have focused on imaging features and degree of enhancement on multiphasic, multidetector CT or MRI as a means of distinguishing AML with minimal fat from RCC. There have been reports [8], [9], [11], [18]–[24] of subtype differentiation of RCCs by CT or MRI.

Perfusion, which is a functional parameter, is the process of a body delivering blood to a capillary bed in its biological tissue. Perfusion is the amount of blood that passes through each unit volume of a tissue. As the metabolic requirements of the tissues are supplied through perfusion, perfusion examination is the indirect criterion of the tissue’s metabolic activity. Perfusion imaging redefines CT as a technique that can now depict vascular physiology in addition to detailed anatomy [25], [26]. Tissue perfusion may be estimated on a segmental basis by calculating time-density curves from dynamic CT acquisitions [27]. Currently, the major applications of perfusion CT are in acute stroke and oncology. Within the kidney, not only can perfusion CT measure alterations of glomerular filtration [28] and assess angiogenesis [14], [29], but it has also been used to demonstrate reduced renal perfusion in renal vein thrombosis, renal artery stenosis, renal obstruction, and cyclosporine toxicity [26], [27], [30], [31]. Perfusion CT can also be applied to the monitoring of a renal tumor’s response to therapy [32]–[35].

To our knowledge, 320-slice dynamic volume CT perfusion has not previously been used in the differential diagnosis of clear cell RCC, papillary RCC, chromophobe RCC, and AML with minimal fat. Thus, the aim of our study was to assess the value of this modality for the differential diagnosis these renal tumors.

## Materials and Methods

### Patients

This prospective study was approved by the Institutional Review Board of Shanghai Changhai Hospital Ethics Committee. Signed written informed consent was obtained from all participants.

From October 2011 to January 2013, 101 patients with suspected RCC by conventional CT at our department were scheduled for surgical resection and were thus screened for study inclusion. None of the patients had seriously impaired function of the heart, liver, kidneys, and history of allergies to iodine contrast medium. Seven patients were excluded because of unwillingness to participate in the study. Thus, a total of 94 patients underwent renal CT perfusion imaging. All of these cases were confirmed by surgical resection and pathological analysis. Nine masses were excluded from the study at pathologic analysis owing to carcinoma of renal pelvis (n = 4), unclassified RCC (n = 4), and metastatic tumor (n = 1). Finally, the left 85 patients (66 clear cell, seven papillary, five chromophobe, and seven AML with minimal fat) were included in our study. The 7 patients with AML had undergone nephrectomy because AML was not diagnosed on the basis of preoperative CT findings; the tumors had instead been diagnosed at preoperative CT as RCCs. Intratumoral fat cannot be visualized in these tumors on CT imaging, and therefore these tumors were considered minimal-fat AML [6]. The 85 cases comprised 63 males and 22 females with a mean age of 52 years (range 22–76 years) (Table 1).

**Table 1:** Characteristics of Patients.

Characteristic		Clear cell(*n* = 66)	Papillary(*n* = 7)	Chromoph-obe(*n* = 5)	AML withminimal fat(*n* = 7)	RCC(*n* = 78)	Renal tumor(*n* = 85)
Sex*	Male	51 (77)	5 (71)	3 (60)	4 (56)	59 (76)	63 (74)
	Female	15 (23)	2 (29)	2 (40)	3 (44)	19 (24)	22 (26)
Age (y)†		53±10	53±16	45±11	50±11	53±11	52±11

* Data are numbers of patients, and data in parentheses are percentages.

† Data are mean ± standard deviation.

### Equipment and Contrast Agents

All patients were examined with 320-slice dynamic volume CT (Aquilion ONE; Toshiba Medical Systems, Ottawara, Japan). For perfusion imaging, 30–40 ml of iopamidol (Niopam; Ultravist 370, 370 mg/ml; Bracco S.P.A., Italy) was injected through a 20-gauge needle in the antecubital vein at a flow rate of 6.5 ml/s, followed by 30 ml saline solution at the same flow rate. The total duration of injection was about 10 s. A CT power injector (Ulrich Medical, Germany) was used in all cases.

### Procedure Design and Scanning Techniques

We established a dynamic CT protocol with the following parameters: 100 kV tube voltage; 100 mA tube current; 0.5 s gantry revolution time; 1 mm pixel spacing; 512×512 pixel (spatial resolution); and 0.5 mm reconstructed slice thickness. Scanning was performed as follows. Patients were required to breathe in and out lightly and naturally. An abdominal belt was applied to reduce respiratory artifacts. First, an unenhanced single image was obtained to ensure that the kidneys would be completely covered by the 16-cm imaging field. Second, dynamic volume CT scanning was initiated after the delay time, which was 8 s after the beginning of the contrast agent injection. In total, 24 CT volumes of the kidneys were acquired. Each of the 24 scans took 0.5 s (one volume acquisition equals a single gantry rotation at a speed of 0.5 s per 360°rotation). The different temporal sampling intervals were 1.5 s**–**4.5 s, and the process took 79.75 seconds overall.

### Image Post-processing

**Figure 1:**
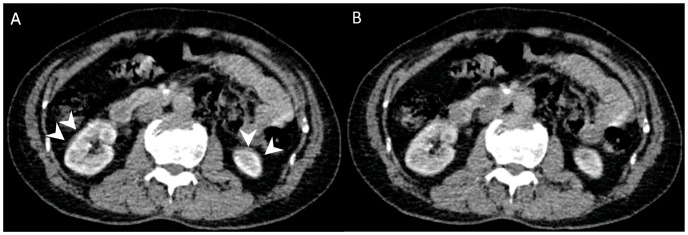
Transverse contrast-enhanced CT scan showed the effect of motion correction. Motion artifacts were seen as blurring of the kidney contours (arrowheads in A). Most motion artifacts were not seen after correction (B).

**Figure 2:**
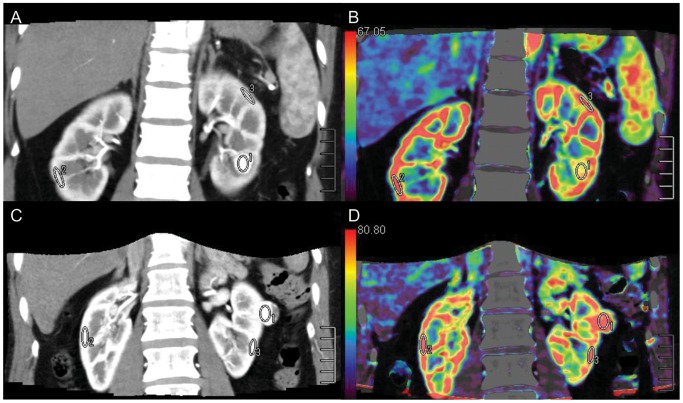
ROIs (ROI 1, ROI 2, and ROI 3) were placed in the tumor and in healthy ipsi- and contralateral renal cortex. ROI 1 was the ROI of tumor tissue. AML with minimal fat (A) (mean CT value = 255.4 HU) with a sufficient blood supply had an abnormal enhancement pattern that was similar to clear cell RCC (C) (mean CT value = 251.4 HU), while Equiv BV was much lower in AML (B) (mean Equiv BV = 57.7) than in clear cell RCC (D) (mean Equiv BV = 85.2).

**Figure 3:**
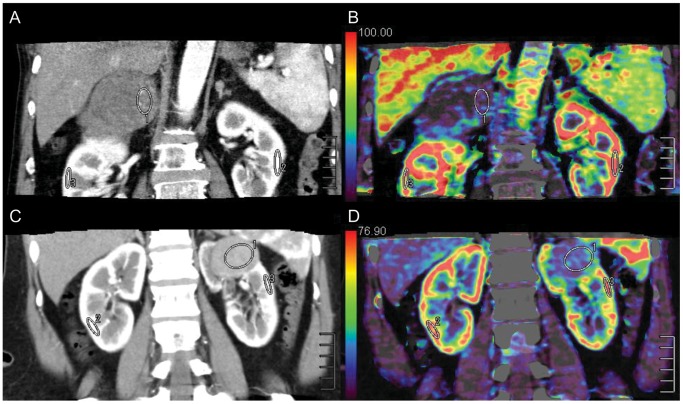
ROIs (ROI 1, ROI 2, and ROI 3) were drawn in the tumor and in healthy ipsi- and contralateral renal cortex. ROI 1 was the ROI of tumor tissue. Papillary RCC (a) with the characteristics of hypo-vascularity (mean CT value = 67.4 HU) showed low perfusion values (mean Equiv BV = 14.1) (B), while chromophobe RCC (C) with a moderate blood supply (mean CT value = 85.0 HU) appeared low perfusion values (mean Equiv BV = 20.8) (D) too.

To correct for motion and breathing differences in all three planes (axial, coronal, and sagittal), image registration (Body Registration, Vitrea fx ves 6.0, nonrigid registration based on global FOV, Toshiba Medical Systems) was performed as the first step. Figure 1 showed an example for image quality before and after the image registration process. Second, the 24 image datasets were post-processed using Body Perfusion software (Vitrea fx ves 6.0, Liscensed software, Toshiba Medical Systems) as followed. The perfusion maps (Figure 2, 3) were generated by Body Perfusion software, and the section thickness of the maps was 5 mm. To optimize visualization of the soft tissue, a processing threshold (CT value range) of between –30 and 400 HU was chosen [14] (Figure 2, 3), and the analysis matrix and noise elimination level were chosen as 128 and strong, respectively. An arterial input was defined within the abdominal aorta by using a mouse to place a circular region of interest (ROI). ROIs of renal tumor and normal renal cortex were defined manually in two planes (axial and coronal). Moreover, ROIs were placed in two different sections of each of the two planes. We took care to ensure that the tumor ROI remained within the internal structure of the mass and to exclude necrosis, cysts, hemorrhage, calcification, and adipose tissue [14] (Figure 2A, 3C). Mean values for perfusion parameters were derived by software and recorded for each patient.

Perfusion was determined using the single input maximal method [36] and Patlak plotting [37]. Based on the aorta and tissue time–density curve, blood flow (BF) was derived by the maximal slope method. Equivalent blood volume (Equiv BV) and permeability surface-area product (PS; clearance/unit volume = permeability) were obtained from a Patlak plot.

### Effective Radiation Dose Evaluation

The effective radiation dose of the protocol was estimated by multiplying the dose–length product from the protocol with the conversion coefficient for the abdomen of 0.015 mSv×mGy^–1^×cm^–1^ [38].

### Statistical Analysis

Quantitative data were expressed as the mean ± standard deviation. The age and sex distribution were compared between the RCC and AML with minimal fat by using the independent sample t test and Chi-square test. Meanwhile, the age and sex distribution were compared between the four different pathologic types tumor by Kruskal–Wallis test and Chi-square test. When continuous values showed a normal distribution, a paired-samples *t*-test was used to compare perfusion parameters between normal cortex and tumor, and an independent-samples *t*-test was used to compare RCC and AML. When values did not show a normal distribution, a two related-samples test (Wilcoxon Signed Ranks Test) and the Mann–Whitney *U* test were used. The Kruskal–Wallis test was used to analyze statistical differences in perfusion parameters between the four different pathologic types of tumor. When there were significant differences in a perfusion parameter among the four different pathologic types, the Nemenyi test was used for multiple comparisons. To evaluate the diagnostic validity of the perfusion values, we analyzed receiver-operating characteristic (ROC) curves and the cut-off values for differentiation of these pathologic types of kidney tumor. Statistical analysis was performed using commercially available software (IBM SPSS 20). *P*<0.05 was considered to indicate statistical significance.

## Results

Clear cell RCC, papillary RCC, and chromophobe RCC accounted for 85%, 9%, and 6% of RCCs, respectively. There were no significant differences in the sex and age distribution of the four different pathologic types of tumor (*P*>0.05). There were no significant differences in the sex and age distribution between RCC and AML with minimal fat (*P*>0.05).

Mean perfusion CT parameter values (Equiv BV, PS, and BF) for normal renal cortex and the renal tumors are summarized in Table 2. The comparison among the perfusion parameters were shown in Table 3. There were significant differences in all the three parameters between tumor and normal renal cortex (*P*<0.001). Equiv BV differed significantly between RCC and AML with minimal fat (*P* = 0.038) (Fig. 2). There were significant differences in Equiv BV and BF among the four different pathologic types of tumor (*P*<0.001 for both). However, Mean Ps (clearance) and BF were similar between RCC and AML with minimal fat (*P*>0.05). No significant difference was found in Ps (clearance) among the four different pathologic types of tumor (*P*>0.05). Table 4 demonstrated the comparison among the four different pathologic types of tumor by the Nemenyi test. Mean Equiv BV and BF were significantly higher in clear cell RCC than in papillary RCC (*P*<0.001 for both) and mean Equiv BV was higher in clear cell RCC than in chromophobe RCC (*P*<0.001). A significant difference was found between the mean Equiv BV of clear cell RCC and that of AML with minimal fat (*P*<0.001). However, Mean BF of clear cell RCC was similar to that of chromophobe RCC (*P* = 0.05). Clear RCC was not significantly different from AML with minimal fat in BF (*P* = 0.63). There were no significant differences in all the two parameters (Equiv BV and BF) among papillary RCC, chromohpobe RCC, and AML with minimal fat (*P*>0.05) (Figure 3).

**Table 2:** Perfusion CT parameter values.

Perfusion parameter	Clear cell(*n* = 66)	Papillary(*n* = 7)	Chromo -phobe(*n* = 5)	AML with minimalfat (*n* = 7)	RCC(*n* = 78)	Renal tumor(*n* = 85)	Normal renal cortex(*n* = 85)
Equiv BV (ml/100 g)	76.6±23.9	28.8±8.6	36.2±16.9	49.3±10.6	69.7±25.3	68.0±25.0	97.8±25.0
Ps (clearance) (ml/100 g/min)	96.8±67.0	58.2±34.2	52.5±19.3	94.1±31.9	90.5±64.2	90.8±62.1	208.4±98.1
BF (ml/100 g/min)	235.2±105.3	74.5±42.9	120.6±86.8	191.7±93.9	213.4±112.3	211.7±110.6	305.4±65.2

Data are mean±standard deviation.

**Table 3:** Comparison among the perfusion parameters.

Perfusion parameter	Normal renal cortex vs renal tumor		RCC vs AML with minimal fat		Four different pathologic types
	Mean difference (95% CI)	*P* value	Mean difference (95% CI)	*P* value	*P* value
Equiv BV	24.8 (−35.1 – −24.4)	<0.001	20.4 (1.2–39.6)	0.038	<0.001
Ps(clearance)	60.0 (−130.5 – −104.7)	<0.001	−3.63 (−52.6–45.4)	0.883	0.147
BF	102.1 (−115.7 – −71.7)	<0.001	21.7 (−65.5–108.9)	0.622	<0.001

The paired-samples *t*-test was used to compare perfusion parameters between normal cortex and tumor, and an independent-samples *t*-test was used to compare RCC and AML with minimal fat.

The Kruskal–Wallis test was used among the four different pathologic types of tumor.

**Table 4:** Comparison among the four different pathologic types of tumor by the Nemenyi test.

Perfusion parameter	Clear cell vs papillary		Clear cell vs chromophobe		Clear cell vs AML with minimal fat		Papillary vs chromophobe		Papillary vs AML with minimal fat		Chromophobe vs AML with minimal fat	
	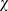 ^2^ value	*P* value	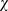 ^2^ value	*P* value	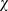 ^2^ value	*P* value	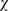 ^2^ value	*P* value	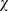 ^2^ value	*P* value	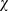 ^2^ value	*P* value
Equiv BV	20.26	<0.001	11.72	<0.001	10.75	<0.001	0.12	0.94	0.83	0.66	0.24	0.89
BF	16.10	<0.001	5.92	0.05	0.91	0.63	0.63	0.73	5.17	0.08	1.64	0.44

**Figure 4:**
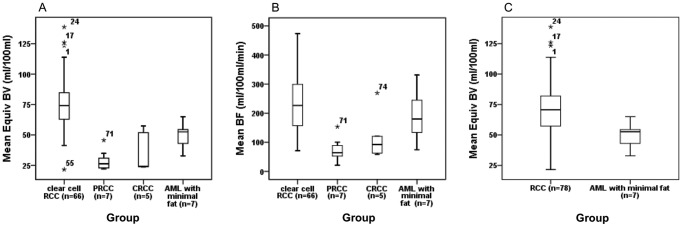
The mean Equiv BV of clear cell RCC was significantly higher than that of papillary and chromophobe tumors and AML with minimal fat (A). The mean BF of papillary RCC was significantly higher than that of clear cell RCC (B). The mean Equiv BV of AML with minimal fat was significantly lower than that of RCC (C). The PRCC and CRCC were the abbreviation for “papillary RCC” and “chromophobe RCC” respectively. *Data were outliers.

The box and whisker plots about the perfusion parameters of different pathologic types of tumor were presented in Figure 4. The results of ROC curve analysis for comparison among the four different pathologic types of tumor were illustrated in Table 5. In addition, the effective radiation dose of the CT perfusion protocol was 18.5 mSv.

**Table 5:** Comparison among the four different pathologic types of tumor by using ROC analysis.

	RCC vs AML withminimal fat(Equiv BV)	Clear cell vspapillary(Equiv BV)	Clear cell vspapillary (BF)	Clear cell vschromophobe(Equiv BV)	Clear cell vs AML withminimal fat(Equiv BV)
Area under ROC curve	0.80	0.98	0.96	0.96	0.92
Threshold value	56.16	47.22	153.84	57.45	56.16
Sensitivity	0.78	0.97	0.79	0.86	0.909
Specificity	0.86	1.00	1.00	1.00	0.86
Accuracy	0.79	0.97	0.96	0.96	0.90

## Discussion

Assessment of organ or tumor perfusion has been of interest for years [39]. In the brain, for instance, CT perfusion imaging plays a clinically important role in detecting ischemic tissue in acute stroke patients [40]. Although perfusion CT has been applied in renal adequacy and other renal diseases [14], [26]–[35], two problems arise with the multidetector-row CT devices in current clinical use. First, because of the limited detector size (current systems from various manufacturers cover 19.2 mm to 4 cm), neither the kidney in its craniocaudal extension nor larger tumors can be imaged in full without table motion during imaging. Second, most patients are unable to hold their breath for longer than 1 min, carrying the risk that the targeted region of the kidney may slip out of the image plane. The newly introduced 320-slice CT device covers an anatomical region of 16 cm in the isocenter of the gantry, which comfortably includes the upper abdominal organs, thus solving the problem of incomplete coverage of both kidneys. The second problem of differences between multiple acquisitions due to patient breathing necessitates the use of registration techniques that virtually eliminate artifacts arising from organ motion [41]. In our study, ROIs of renal tumor and normal renal cortex were defined manually in two planes (axial and coronal). This protocol takes into account the entire tumor, which appears as if the perfusion parameters were measured in three-dimensional volumes of interest [42].

Chen et al. [14] reported CT perfusion values for normal renal cortex; the average BF of 454.32 ml/100 ml/min was higher than that measured in our study. However, the BF in our study was similar to that of Reiner et al. [42] (305.4 ml/100 ml/min vs 297.1 ml/100 ml/min). Our values for the BF of RCC, clear cell, papillary, and chromophobe tumors appear to differ from previous studies [14], [42]. In addition, our results for these parameters cannot be compared with previous studies because of the differences in units between Equiv BV and BV, and PS (clearance) and K^Trans^ [14], [42]. Thus, we think that these differences between the previous studies (Chen et al.’s and Reiner et al.’s) and our study might be attributed to differences in scan protocols, volume analysis, and post-processing algorithms.

The estimated effective radiation dose of the protocol used in our study was controlled to within 18.5 mSv, which is below the effective radiation dose used for standard CT perfusion (33.6±6.8 mSv) [43].

With the use of an advanced CT scanner and the described CT protocols and post-processing software, we consider the perfusion parameter values obtained in our study to be accurate.

In this study, all of the perfusion parameter values for tumor were significantly lower than those for normal renal cortex, which is in agreement with the recent studies of Chen et al. and Reiner et al. [14], [42]. Our finding that Equiv BV and BF were significantly higher in clear cell RCC than in papillary RCC is in line with that of Chen et al. [14]. These results might be explained by alterations of microvessel architecture in tumor [44].

Similar to clear cell renal carcinoma, AML with minimal fat also appears to exhibit abnormal enhancement with a “fast-in-and-fast-out” pattern during the early phase that is easily misdiagnosed as renal cancer. It is difficult to differentiate them merely through CT [45]. Kimet al. [6] compared various CT features of AML with minimal fat with those of size-matched RCCs and found that biphasic helical imaging may be useful in differentiating AML with minimal fat from RCC, with homogeneous tumor enhancement and prolonged enhancement pattern being the most valuable CT findings. Sasiwimonphan et al. [17] thought that a combination of T2 signal intensity (SI) ratio less than 0.9 with SI index greater than 20% plus T1 SI ratio greater than 1.2 or arterial-to-delayed enhancement ratio greater than 1.5 was accurate in differentiating AML from RCC. We found mean Equiv BV to be significantly lower in AML with minimal fat than in clear cell RCC and in RCC. To the best of our knowledge, such findings have never been reported in previous studies. Zhang et al. [15] reported that the unenhanced attenuation characteristic, intratumoral vessels, and the attenuation values of unenhanced and early excretory phase scans are valuable parameters in differentiating AML with minimal fat from papillary RCCs on CT. We found no significant difference between the Equiv BV, PS (clearance), and BF of non-clear cell RCC and AML with minimal fat, which might be attributed to the limitations of this study. There were insufficient numbers of cases of non-clear cell RCC and AML with minimal fat, so further studies are needed.

Some investigators [8], [9], [11], [18]–[24] have focused on the use of imaging features, most notably degree of enhancement on multiphasic, multidetector CT, as a means of distinguishing RCC subtypes. Their studies have shown that the degree of enhancement of clear cell RCC is greater than that of other RCC subtypes. In our study, mean Equiv BV and BF were significantly higher in clear cell RCC than in papillary RCC and mean Equiv BV was higher in clear cell RCC than in chromophobe RCC. There was no significant difference in Equiv BV, PS (clearance), or BF between papillary and chromophobe RCC.

In our study, clear cell RCC, papillary RCC, and chromophobe RCC accounted for 85%, 9%, and 6% of RCCs, respectively. The distribution in our study as well as another study (only 4% of the cases had papillary RCC) [14] did not match the distribution in other studies [10], [11]. We think that this difference is attributable to the difference in the race of the study populations and the small sample size. Additionally, further studies are needed to clarify this.

There were limitations to our study. First, our CT protocol delivered a radiation dose about two times higher than that delivered by diagnostic CT used to image anatomy. Protocols that reduce this radiation exposure deserve further study. Second, the study included only a limited number of papillary and chromophobe RCCs, so accurate evaluation of the perfusion features of these pathologic types was not possible. Finally, we did not evaluate the correlation between perfusion CT parameters and renal tumor angiogenesis.

In summary, perfusion imaging by 320-slice dynamic volume CT can be used to measure hemodynamic features of the whole kidney and of kidney tumors, which may be useful in the differential diagnosis of these four pathologic types of kidney tumor.

## Supporting Information

Figure S1 **Comparative 3-dimensional (3D) rendered images.** Axial (A), coronal (B) and sagittal (C) contrast-enhanced CT showed an AML with intratumoral fat in the left kidney. An AML with minimal fat of the left kidney was illustrated in Axial (D), coronal (E) and sagittal (F) contrast-enhanced CT.
(TIF)
