# Supplementary figures and images for: Study of 320-Slice Dynamic Volume CT Perfusion in Different Pathologic Types of Kidney Tumor: Preliminary Results
Source: PLoS One. 2014 Jan 21;9(1):e85522. doi: 10.1371/journal.pone.0085522 (PMC3897451; doi:10.1371/journal.pone.0085522)

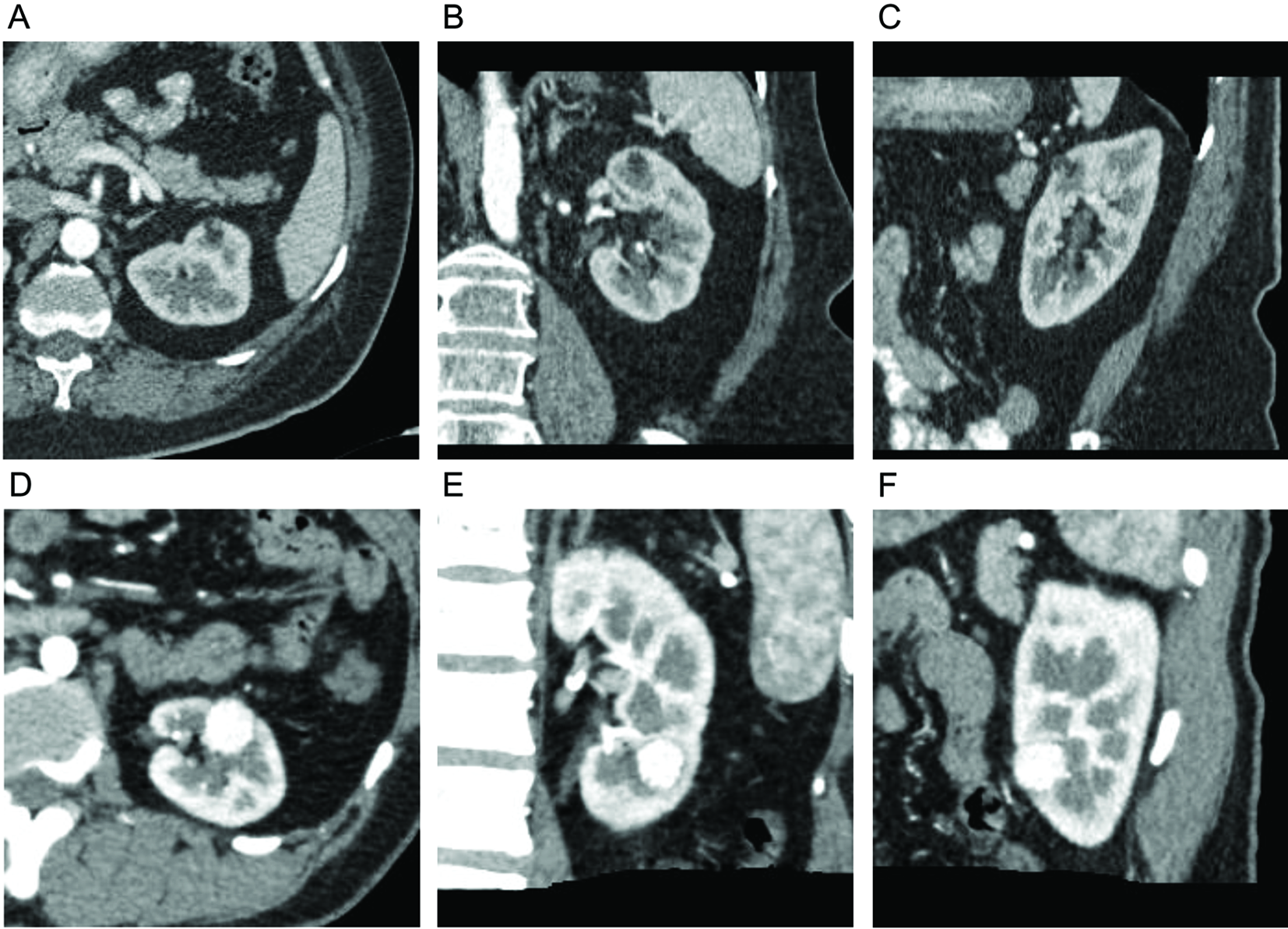

Supplement: Figure S1 — Comparative 3-dimensional (3D) rendered images. Axial (A), coronal (B) and sagittal (C) contrast-enhanced CT showed an AML with intratumoral fat in the left kidney. An AML with minimal fat of the left kidney was illustrated in Axial (D), coronal (E) and sagittal (F) contrast-enhanced CT. (TIF) [file pone.0085522.s001.tif]
